# Loss of a Fragile Chromosome Region leads to the Screwy Phenotype in *Paramecium tetraurelia*

**DOI:** 10.3390/genes10070513

**Published:** 2019-07-06

**Authors:** Irina Nekrasova, Vera Nikitashina, Simran Bhullar, Olivier Arnaiz, Deepankar P. Singh, Eric Meyer, Alexey Potekhin

**Affiliations:** 1 Department of Microbiology, Faculty of Biology, Saint Petersburg State University, Universitetskaya nab. 7/9, 199034 Saint Petersburg, Russia; 2 IBENS, Département de Biologie, Ecole Normale Supérieure, CNRS, Inserm, PSL Research University, F-75005 Paris, France; 3 Institute for Integrative Biology of the Cell (I2BC), CNRS, CEA, Univ. Paris-Sud, Université Paris-Saclay, 91198 Gif-sur-Yvette CEDEX, France; 4 Friedrich Miescher Institute for Biomedical Research, Maulbeerstrasse 66, 4058 Basel, Switzerland

**Keywords:** *Paramecium*, micronuclear deletion, chromosome fragile sites, cortical inheritance, trichocysts, epimutation, maternal inheritance

## Abstract

A conspicuous cell-shape phenotype known as “screwy” was reported to result from mutations at two or three uncharacterized loci in the ciliate *Paramecium tetraurelia*. Here, we describe a new screwy mutation, Spinning Top, which appeared spontaneously in the cross of an unrelated mutant with reference strain 51. The macronuclear (MAC) genome of the Spinning Top mutant is shown to lack a ~28.5-kb segment containing 18 genes at the end of one chromosome, which appears to result from a collinear deletion in the micronuclear (MIC) genome. We tested several candidate genes from the deleted locus by dsRNA-induced silencing in wild-type cells, and identified a single gene responsible for the phenotype. This gene, named Spade, encodes a 566-aa glutamine-rich protein with a C_2_HC zinc finger. Its silencing leads to a fast phenotype switch during vegetative growth, but cells recover a wild-type phenotype only 5–6 divisions after silencing is stopped. We analyzed 5 independently-obtained mutant alleles of the *Sc1* locus, and concluded that all of them also lack the Spade gene and a number of neighboring genes in the MAC and MIC genomes. Mapping of the MAC deletion breakpoints revealed two different positions among the 5 alleles, both of which differ from the Spinning Top breakpoint. These results suggest that this MIC chromosome region is intrinsically unstable in strain 51.

## 1. Introduction

The ciliate *Paramecium tetraurelia* is one of a few traditional objects of genetic studies of unicellular eukaryotes [1]. Sex occurs through one of two different processes: conjugation, where two partners exchange gametic pronuclei to produce a pair of genetically-identical heterozygous progeny, and autogamy, a self-fertilization process resulting in entirely homozygous progeny. These features are highly advantageous for genetic analysis. Like all ciliates, *P. tetraurelia* contains two types of nuclei in each cell. The germline, diploid micronucleus (MIC) acts as a silent transmitter of chromosomes across sexual generations, while the somatic, highly polyploid macronucleus (MAC) is responsible for all gene expression. The extensive programmed genome rearrangements that occur during the development of a new MAC from the zygotic nucleus in each sexual generation have led ciliates to become recognized models for epigenetics [2,3].

**Figure 1:**
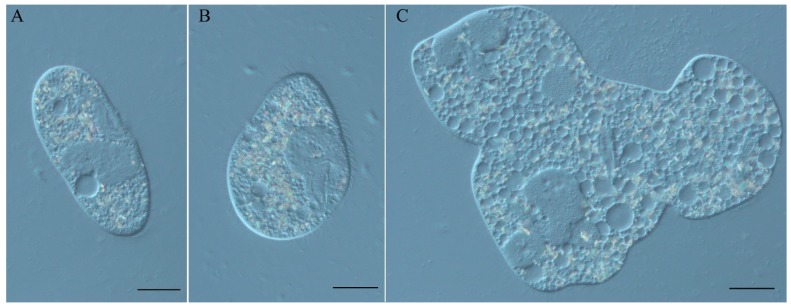
(**A**) *P. tetraurelia* strain 51 wild type cell; (**B**) Spinning Top mutant cell; (**C**) Spinning Top multinuclear monster cell. DIC, Nikon Eclipse Ni-U microscope (Nikon, Japan). Scale bar is 20 µm.

In this work, we studied the Spinning Top mutant of *P. tetraurelia* strain 51, which appeared spontaneously in a back-cross of the unrelated mutant *mtGa-1* [4] to the wild type. This mutant has a remarkable phenotype (Figure 1), which is characterized by short pear-shaped cells quickly spinning around their pointed anterior tip when swimming (Video S1 and Video S2). Sometimes such cells lose the ability to complete fissions (Figure 1C).

In a summary of all available data on *P. tetraurelia* mutants, Sonneborn [5] mentioned mutations at two or three loci that result in very similar “screwy” phenotypes. The *sc1* and *sc66* loci were shown to be unlinked; the *sc66* locus was found to segregate independently from *sc1*, but no complementation or linkage test was performed with *sc64*, so it may or may not represent a third locus. At least 10 alleles of the best-documented *sc1* locus result in “cells of variable body shape: round or pear-shaped and extremely twisted like a cork-screw, when grown with excess food; when starved, shape approaches normal, but is somewhat bent, like cashew nut. Corkscrew-shaped cells rotate rapidly on their long axis during locomotion” [5]. Mutant alleles were classified in 4 groups. *sc1-a*, *sc1-b* and *sc1-d* alleles yield normal carrot-shaped trichocysts, while alleles Sonneborn grouped as *sc1-c* have cigar-shaped (but functional) trichocysts, thinner and often longer than normal trichocysts, and do not taper to the same degree [6]. Most screwy mutants are characterized by slow growth and the tendency to produce monsters due to incomplete fissions [5,7,8]. Thus, many screwy mutations are pleiotropic.

Several *sc1* alleles obtained independently in different studies (Table 1) were available from the stock collection of the Centre de Génétique Moléculaire in Gif-sur-Yvette, France. In this work we figured out that the Spinning Top mutation is allelic to *sc1* mutations. All alleles examined showed rather large MAC deletions encompassing several genes, which appear to reflect similar deletions in the germline MIC genome. We further identified the one gene at this locus that is responsible for the screwy phenotype, and analyzed the patterns of inheritance of the discovered deletions.

## 2. Materials and Methods

### 2.1. Paramecium Strains, Cultivation, and Genetic Analysis

Paramecia were grown in a wheat grass powder (WGP; Pines International, Lawrence, KS, USA) infusion medium bacterized the day before use with *Klebsiella pneumoniae*, and supplemented with 0.8 mg/L of β-sitosterol (Merck, Darmstadt, Germany). Cultivation, synchronization of cultures and autogamy were carried out by daily reisolations at 27 °C (as described in [10]). *P. tetraurelia* strain 51 was used as the wild-type strain in back-crosses and gene silencing experiments. All available screwy mutants (Table 1) originating from strain 51 or from its largely isogenic derivative d4–2 used in the study were maintained in the stock collection of the Centre de Génétique Moléculaire in Gif-sur-Yvette, France. All currently extant strains are available upon request from that collection or from CCM collection (Saint Petersburg State University, Saint Petersburg, Russia).

All studied mutant strains were shown to be mating type O by conjugation with *P. tetraurelia* tester strains. Mating type tests and crosses were performed by a standard approach [10]. F1s were obtained by conjugation, F2s were derived from F1 clones by autogamy. A trichocyst non-discharge recessive mutation (nd7) introduced into wild-type strain 51 of mating type E was used as a genetic marker in crosses. Exchange of pronuclei in conjugating couples was confirmed by checking the phenotypes of F1 clones; those derived from couples where the mitotic progeny of both exconjugants were able to discharge trichocysts treated with picric acid were considered as true heterozygotes.

### 2.2. Spinning Top Mutant Macronuclear Genome Sequencing and Analysis

Sixty F2s from one F1 heterozygote from Spinning Top x wild type 51 cross were obtained. Fifty-eight F2 clones survived; 29 of them were wild-type and 29 were Spinning Tops. The latter were pooled, and the total DNA sample of the mutant pool was prepared. Genome sequencing details can be found in [4]; dataset accession number: ERS2516654.

### 2.3. RNAi-Mediated Gene Silencing by dsRNA Feeding

Plasmids used for T7Pol-driven dsRNA production in silencing experiments were obtained by cloning PCR products from each gene using plasmid L4440 and *E. coli* strain HT115 DE3, as previously described [11]. Genes used in silencing experiments are indicated in Table S1. dsRNA feeding media were prepared using WGP medium as described [12]. Silencing in vegetative cells was achieved by manual isolation of 100 paramecia to dsRNA feeding medium. After 12 hours of incubation in silencing media if the phenotype switch was observed the cells were transferred back to conventional WGP medium, and the reversion to the wild type phenotype was monitored. The procedure to knockout genes during autogamy has been described before [12]. Postautogamous cells were isolated after 3–4 days into conventional WGP medium and further maintained routinely.

### 2.4. Localization of the Chromosome Breakage Point in the Mutant Strains

The presence/absence of genes from the Spinning Top locus in the MAC genomes of the screwy mutants was verified by PCR of different regions of that locus. DNA from the strains studied was isolated with NucleoSpin^®^ Tissue kit (Macherey-Nagel, Germany) according to the protocol Genomic DNA from tissue. Encyclo Taq polymerase (Evrogen, Russia) was used for PCR, the primers were synthesized by Beagle (Russia) and Eurofins DNA (Germany). In order to localize precisely the breakage point within the MAC genome scaffold 77 we applied the chromosome walking method starting from the deletion site in Spinning Top mutant downstream to 3’-end of scaffold 77 (designed primers are listed in Table S2). When no PCR product was obtained it meant that a downstream primer was not finding homology in the mutant genome. DNA of wild type strain 51 was used as a template in PCR positive control. PCR without template DNA was always performed as negative control.

To figure out if the discovered deletion occurred in MAC genome or in MIC genome of Spinning Top mutant and screwy mutants, MIC-specific PCR was performed, when one PCR primer was located within IES (internal eliminated sequence) apparently absent from the MAC genome. The primers (Table S2) were designed to include another IES within the amplified region; PCR products were cloned (CloneJET PCR Cloning Kit, Thermo Scientific, USA, was used) and sequenced. The presence of the IES inside the sequence confirmed that such PCR was indeed utilizing MIC but not MAC DNA as a template. A fourfold concentration of total template DNA in PCR mix compared to routine PCR was sufficient to achieve successful MIC PCR. Sequencing was performed in the Core Facility Center “Molecular and Cell Technologies” (St Petersburg State University, Saint Petersburg, Russia) and GATC Biotech (Cologne, Germany).

## 3. Results

### 3.1. Characterization of the Spinning Top Mutation

**Figure 2:**
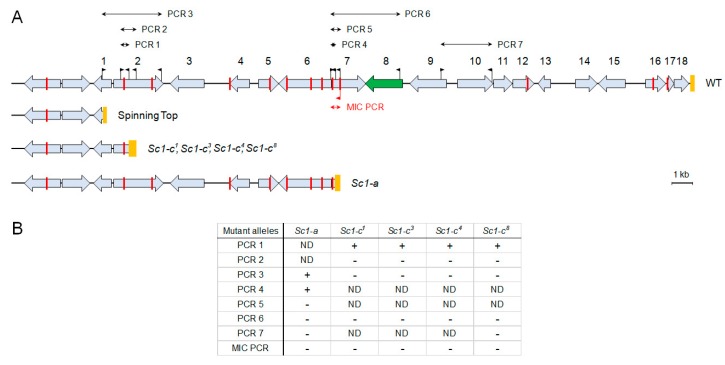
(**A**) Genomic map of *P. tetraurelia* strain 51 MAC scaffold 77 right end (WT), and localization of three inferred deletions in the studied screwy mutants. The genes are shown as grey boxes with arrows; deleted in Spinning Top segment encompasses those with numbers. The *Spade* gene box is colored with green. Orange boxes indicate the telomere addition regions for the WT and Spinning Top; for other mutants they represent the regions in which the deletion breakpoints were mapped. The flags correspond to the primers used in chromosome walking PCRs and for MIC-specific PCR. Red bars indicate the positions of IES excision junctions in the MAC sequence. The red flag below the line is a MIC-specific primer within an IES. (**B**) Summarized results of all PCR tests in all mutants (+ stands for PCR product present, - stands for PCR product absent, ND – not done). For the list of the PCRs and the primers used see Table S1).

The Spinning Top mutant appeared spontaneously as one of 56 wild-type (*mtGa*/*mtGa*) homozygotes obtained by autogamy of an *mtGa*/*mtGa-1* heterozygote [4] (Figure S1); none of the other F2 homozygotes, whether wild-type or mutant for *mtGa*, showed the same screwy phenotype. When that Spinning Top clone was crossed to the wild type, all true F1s showed a wild-type phenotype. In postautogamous F2 sets obtained by autogamy of six F1 clones from both cytoplasmic origins, a clear segregation of wild-type and Spinning Top phenotypes was observed among F2 clones after several divisions (the ratio in the initial experiment was 29:29, and repeated genetic analyses confirmed that it is always close to 1:1). Thus, the Spinning Top phenotype appears to be due to a new Mendelian (i.e., micronuclear) mutation, which was not present in the original *mtGa-1* mutant. The mutated locus was then identified by resequencing the MAC genome from a pool of 29 phenotypically mutant F2 clones, following a previously established procedure [13]. This revealed the deletion of a 28.5-kb segment corresponding to the right end of scaffold51_77 in the wild-type MAC genome [9], which contains 18 genes (Figure 2) according to the latest annotation [14]. Micronucleus-specific PCRs, using primers located in Internal Eliminated Sequences (IESs), indicated that the deletion also occurs in the MIC, over at least several kb after the breakpoint. The Spinning Top Mendelian mutation thus appears to be this MIC deletion, rather than another mutation elsewhere in a trans-acting factor that could cause the abnormal elimination of a wild-type segment of the genome during MAC development.

### 3.2. Spade Gene Identification

**Figure 3:**
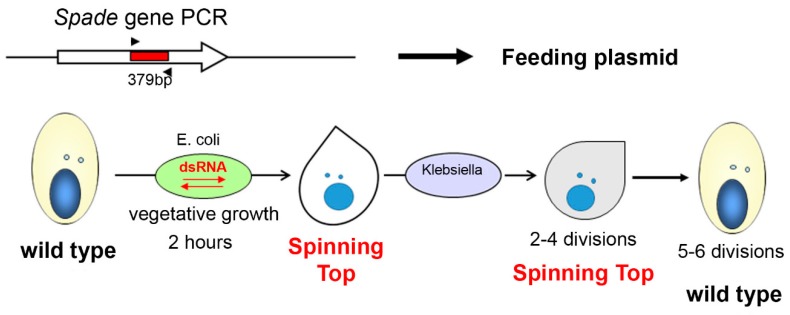
The scheme of *Spade* gene knockdown in vegetative *P. tetraurelia* cells by dsRNA feeding. The PCR amplicon of a target sequence was inserted into a feeding plasmid, which was then used for *E. coli* transformation. Ciliates feeding on transformed *E. coli* stop expressing the target gene until silencing is over. Analogous procedure was applied for copine and NimA genes, and had no phenotypic effect.

At least one of the 18 genes missing in the Spinning Top MAC genome (Table S3) can be expected to be responsible for the screwy phenotype. To identify this gene, we selected several candidate genes in the deleted locus and tested their function by dsRNA-induced silencing. One of the genes (PTET.51.1.G0770200) encodes a copine, a type of membrane-associated calcium-binding proteins which are probably involved in membrane trafficking in different organisms [15]. Another one (PTET.51.1.G0770206) encodes a 566-aa glutamine-rich protein with a zinc finger of the C_2_HC type and a coiled-coil domain. The third one (PTET.51.1.G0770208) encodes a protein kinase which was provisionally assigned to the NimA (Never In Mitosis gene A)-related kinase family Nek. These kinases have several basic roles in cell biology, and mutations of Nek family members have been identified as drivers behind the development of ciliopathies [16]. There were at least three whole genome duplications in *Paramecium* evolution [17]. The copine gene has a highly conserved ohnolog from the last whole genome duplication (WGD1), making it a less likely candidate since WGD1 ohnologs usually have redundant functions that can mask the effect of a mutation [18]. The other two genes only have more divergent ohnologs from the previous whole genome duplication (WGD2). The candidate genes were switched off by dsRNA-induced silencing in vegetative cells, and in one case, this recapitulated the screwy phenotype. Knockdown of the zinc-finger-containing protein gene led to a fast phenotype switch in vegetative cells, which, after two hours of incubation in the dsRNA feeding medium, were becoming identical to the Spinning Top mutants (Figure 3). Thus, the lack of the PTET.51.1.G0770206 gene appears to be what determines the screwy phenotype in the mutant. This gene was named *Spade*, as the shape of the mutant cells resembles a spade from a deck of cards.

Interestingly, when dsRNA feeding was stopped and cells were transferred back to the usual medium; they did not recover a wild-type phenotype after several hours, as often happens for other genes [11]. Silencing of the *Spade* gene resulted in a metastable phenotype, which did not revert to the wild type until several vegetative divisions after the silencing was stopped. By following the fate of 30 cell lines established from silenced cells, we found that 5–6 divisions, rather than any absolute time period, were necessary for phenotypic reversion. Thus, the cortical disorder induced by *Spade* silencing could only repair itself gradually during cell divisions.

Since the screwy phenotype can result from mutations at several loci, we tested whether the WGD2 *Spade* ohnologs (PTET.51.1.G0810029 and PTET.51.1.G0940176) might be involved in the same function, despite they have no significant homology to the *Spade* gene. dsRNA-induced silencing of each of them, however, did not result in any detectable phenotype. The other *Sc* loci thus remain unknown.

### 3.3. Analysis of Other Screwy Mutants

**Figure 4:**
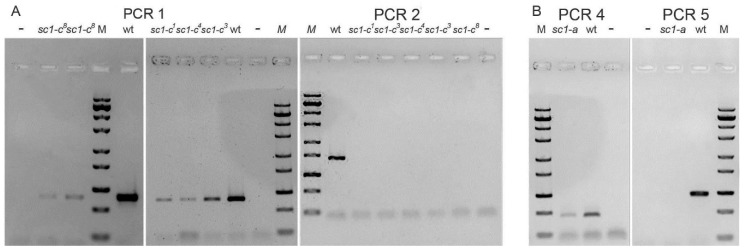
Localization of the breakage sites within MAC scaffold 77 for screwy mutants. (**A**) Results of PCRs framing the chromosome breakage points in allelic mutants *sc1-c*. (**B**) Results of PCRs framing the chromosome breakage point in mutant *sc1-a*. PCR products are not obtained when sequence homologous to the downstream primer is absent (PCR2; PCR5). The products of these PCRs are always present in wild type 51 strain (wt). “–“ stands for negative control. M is ZipRuler™ Express DNA Ladder Set (Thermo Fisher, USA). For primers used in PCRs see Figure 2 (mapping) and Table S1 (sequences).

To determine whether previously obtained screwy mutants are also affected in the *Spade* gene, we used PCR to examine the gene in 5 available mutant strains, all containing different alleles of the *Sc1* locus (*sc1-a*, *sc1-c^1^*, *sc1-c^3^*, *sc1-c^4^*, *sc1-c^8^*). The best part of the *Spade* gene and downstream sequences were amplified by PCR 6 (Figure 2), which did not yield any product from total DNA of any of these strains, indicating that their MAC genomes lack the gene (Figure S2). PCRs for different parts of the locus deleted in Spinning Top indicated that they also lack a number of neighboring genes. Thus, all tested *sc1* alleles show deletions at the right end of MAC scaffold51_77. Walking the chromosome by PCRs allowed us to localize rather precisely the breakage sites for all alleles (Figure 4).

We detected at least two types of deletions which both differ from the 28.5-kb terminal deletion observed in Spinning Top (Figure 2). The first of these, starting approximately 27 kb from the wild-type MAC telomere, was observed for the four *sc1-c* alleles. The upstream breakpoints were all localized within a 345-bp segment of the gene PTET.51.1.G0770200. The second deletion type, starting about 17 kb upstream of the wild-type telomere, was observed for the *sc1-a* allele; the breakpoint was localized within a 262-bp segment of gene PTET.51.1.G0770205. We cannot exclude that the MAC deletions observed in these 5 strains are in fact internal to the chromosome, rather than terminal ones as in Spinning Top, as we did not test the presence of the last genes before the wild-type telomere. However, PCR 7 (Figure 2), which was performed for the *sc1-a* and *sc1-c^8^* alleles, yielded no product, indicating that these MAC deletions extend at least a few kb beyond the *Spade* gene. Assuming that they are all terminal deletions, the *sc1-c* mutants would retain one more gene than Spinning Top, and the *sc1-a* mutant 5 more genes than *sc1-c* mutants.

**Figure 5:**
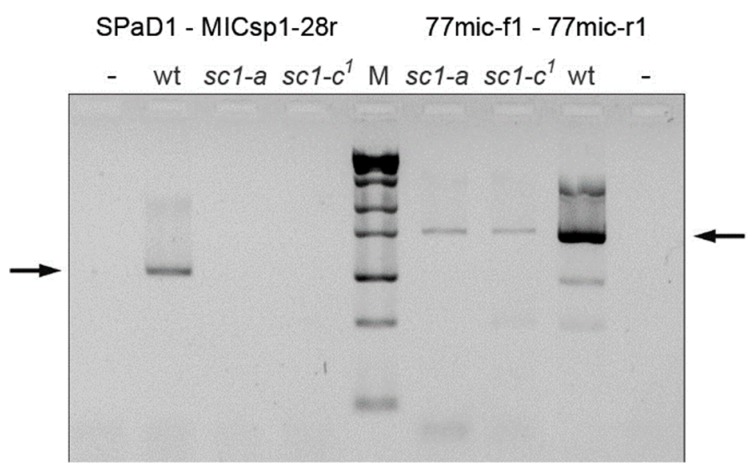
The MIC deletions in screwy mutants *sc1-a* and *sc1-c^1^* confirmed by MIC-specific PCRs. Arrows show the expected PCR products. Control PCR of the region upstream to the deletion yielded the product for both mutants, while PCR product from the region deleted in MAC (left to the marker) is missing in both mutants, thus they lack this region also in their MICs. wt is wild-type strain; “–” stands for negative control; M is ZipRuler™ Express DNA Ladder Set (Thermo Fisher, USA). For primers used in PCRs see Figure 2 (mapping) and Table S1 (sequences).

To determine whether the MAC deletions observed in *sc1-a* and *sc1-c* mutants may be due to collinear deletions in the MIC genome, we performed a MIC-specific PCR targeting a sequence close to the *Spade* gene, using one primer located in an IES sequence (which is excised from the MAC genome). To ensure specificity, the PCR was designed so that the amplified segment would include another IES, allowing specific MIC amplification to be confirmed by sequencing the PCR product (Figure 2 and Figure 5). This was indeed the case when PCR amplification was performed on total DNA from wild-type cells, despite the vast excess of MAC over MIC DNA (200:1) in the sample. When performed in the same conditions on total DNA from the screwy mutants, however, the MIC-specific PCR still yielded no product, suggesting that the target sequence was also absent from their MIC genomes. To make sure that the mutant samples contained sufficient amounts of MIC DNA, a control MIC-specific PCR was similarly designed to target a different region of scaffold51_77, outside of the locus deleted in the MAC of the mutants. The control PCR successfully amplified the target in both wild-type and mutant DNA samples (Figure 5); sequencing of the PCR products confirmed the presence of the test IES in all cases, demonstrating the specificity of MIC amplification. We concluded that the MIC genomes of the *sc1* mutants tested indeed carry deletions of at least part of the *Spade* genomic region, which thus appears to correspond to the genetically defined locus *sc1*. The different MAC deletions observed in different mutants may simply reflect different extents of the MIC deletions in different *sc1* alleles.

### 3.4. Induction and Inheritance of MAC Deletions at the sc1 Locus

The MAC genome is a rearranged version of the MIC genome, and obviously it cannot contain any sequence that is absent from the latter. However, MIC sequences may or may not be retained in the MAC during its development: the rearrangement program was shown to be epigenetically controlled by the parental MAC in each cell through a homology-dependent process mediated by the scnRNA pathway [2,3]. This ensures the developmental elimination of any MIC sequence that was not previously retained in the parental MAC, resulting in the maternal (cytoplasmic) inheritance of alternative rearrangements during conjugation of wild-type cells. We therefore wondered what the developmental fate of the wild-type *sc1* allele would be after it was introduced by conjugation in the zygotic genome formed by fertilization in a mutant cell, where the *Spade* genomic region is absent from the old MAC.

**Figure 6:**
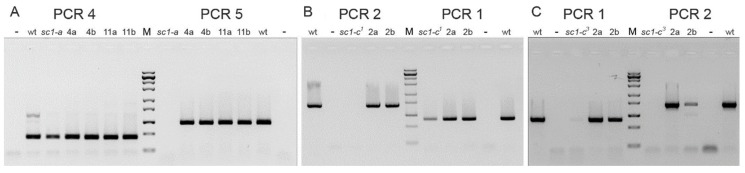
Inheritance of the deletions inferred in screwy mutants. (**A**) Results of PCRs for the cross *sc1-a* x wt (wild type strain 51). 4a, 4b, 11a, 11b are pairs of exconjugant F1 clones from the cross. Both PCR4 primers are present in the mutant genome, while downstream primer of PCR5 is absent. (**B**) Results of PCRs for the cross *sc1-c^1^* x wt. 2a, 2b are a pair of exconjugant F1 clones from the cross. (**C**) Results of PCRs for the cross *sc1-c^3^* x wt. 2a, 2b are a pair of exconjugant F1 clones from the cross. For (**B**,**C**) both PCR1 primers are present in the mutant genome, while downstream primer of PCR2 is absent. M is ZipRuler™ Express DNA Ladder Set (Thermo Fisher, USA); “–” stands for negative control. The PCRs show that the sequence deleted in the mutant parent is always present in both heterozygous F1s (from both cytoplasmic lineages) obtained in a cross of the mutant to the wild-type parent. Thus, deletions in mutant’ *sc1* loci are not inherited maternally, which allows to suggest that they occur in MICs.

To answer this question, we crossed the Spinning Top, *sc1-a*, *sc1-c^1^* and *sc1-c^3^* with the wild-type strain, and examined the processing of the wild-type *sc1* allele during development of zygotic MACs in pairs of F1 heterozygotes, one from each cytoplasmic parent. In all cases, both F1 clones showed a wild-type phenotype, suggesting that the wild-type *sc1* allele was not deleted in the heterozygous MAC developing in the cytoplasm of the mutant parent, under the control of a parental MAC where the locus is deleted. This was confirmed by PCR analyses, which showed that the wild-type *sc1* allele is retained in the MACs of all F1 clones (Figure 6). Thus, the deletion observed in the old MAC of the mutant parent is not epigenetically reproduced during development of the zygotic MAC in this case. While at first sight this may seem to be contradictory with the maternal inheritance of alternative rearrangements in wild-type cells, it is in fact fully consistent with the current model for scnRNA action (see Discussion). Consistently, after autogamy of F1 heterozygotes, we observed 1:1 segregation of wild-type and screwy phenotypes among F2 homozygotes.

**Figure 7:**
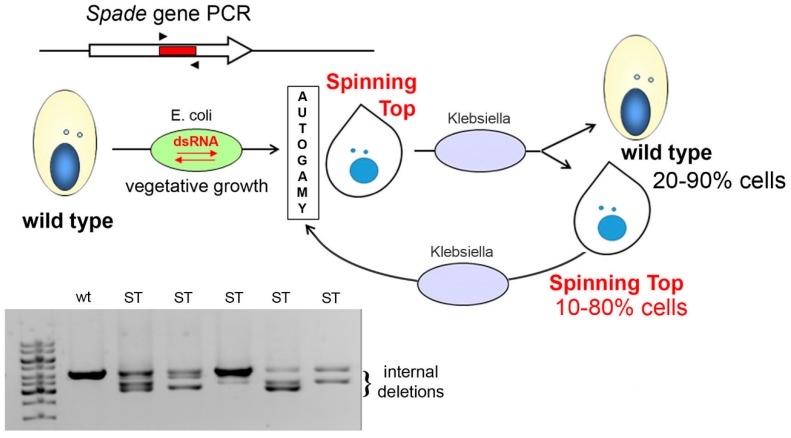
dsRNA feeding during autogamy results in imprecise deletions in the target locus. A segment from the *Spade* gene was inserted into the vector used for dsRNA production in *E. coli*. Cells underwent autogamy in the feeding medium, which resulted in variable ratios of wild-type (wt) and Spinning Top (ST) phenotypes among postautogamous progeny. All postautogamous clones showed internal deletions in at least a fraction of copies of the target locus, as detected by PCR using primers SPaD1 and SPaD2 (Table S2). In each life cycle, after the cells with induced MAC deletions undergo autogamy, they continue to produce wild-type and mutant cells in different ratios in vegetative progeny.

We further checked whether MAC deletions in the *sc1* locus would be maternally inherited in cell lines that have a homozygous wild-type MIC genome. To create such cell lines, we relied on the observation that dsRNA-induced silencing of a gene during autogamy of wild-type cells results in the developmental elimination of that gene in the developing zygotic MAC [19]. We submitted wild-type cells to dsRNA feeding for the copine, *Spade* and NimA genes, either individually or in combinations, and let the cells undergo autogamy in the silencing media. In each case, imprecise deletions of variable lengths encompassing the sequences used for dsRNA feeding were observed in the MACs of post-autogamous progeny, as expected (Figure 7).

In all cases where the *Spade* gene sequence was used for dsRNA feeding, the screwy phenotype appeared with variable intensities and in variable fractions of postautogamous progeny (in total, 23 out of 33 isolated postautogamous clones showed a phenotype that was stable during vegetative growth). dsRNA feeding with copine or NimA sequences was much less efficient at inducing screwy phenotypes (only 4 out of 12 clones showed a transient phenotype which had disappeared after 10 divisions); presumably the distances between these genes and the *Spade* gene result in a smaller fraction of induced deletions affecting the latter. When the *Spade* and NimA genes (located closer to each other than to the copine gene) were targeted together, a higher fraction of postautogamous progeny showed the screwy phenotype.

All clones containing at least a fraction of phenotypically mutant cells after 10 divisions were tested by PCR, and all showed MAC deletions of variable lengths in a fraction of copies of the *sc1* locus (Figure 7, Figure S3a). However, during vegetative growth, phenotypically mutant clones continuously produced cells that reverted to the wild-type phenotype. This was still the case after these clones were maintained in stock tubes for 9 months, which implies several successive rounds of autogamy. To assess the stability of the phenotype through autogamy, we established one phenotypically mutant and one phenotypically wild-type cell from each of three mixed populations. This established 6 cell lines that were then allowed to undergo autogamy, and 10 postautogamous cells were isolated from each of them. None of the 30 postautogamous clones from phenotypically wild-type cell lines showed the screwy phenotype. Among the 30 postautogamous clones from phenotypically mutant cell lines, 22 had entirely reverted to the wild type, and 8 showed at least a fraction of screwy cells. Of these, 2 clones produced only cells with a strong phenotype, while the remaining 6 produced both wild-type and mutant phenotypes in different ratios. Clones containing phenotypically mutant cells still showed variable deletions in a fraction of MAC copies of the *sc1* locus when tested by PCR (Figure S3b). We conclude that MAC deletions at the *sc1* locus can indeed be epigenetically inherited through autogamy in cell lines with a wild-type MIC genome as relatively stable epimutations, although wild-type revertants appear at a high frequency.

## 4. Discussion

In this work we studied the Spinning Top mutation in *P. tetraurelia* and identified the gene leading to the screwy phenotype, which we named *Spade*. The Spinning Top mutation was found to be a rather large (≥28.5 kb) MIC deletion that removes 18 genes, including *Spade*. Several screwy mutants have been described earlier [5], but the affected loci had not been identified molecularly. We analyzed 5 different mutant alleles of the *Sc1* locus which had been independently obtained in previous studies, and found that all of them show deletions of the *Spade* and neighboring genes in their MAC and MIC genomes, though the deletion breakpoints differ among them. The high frequency of deletions observed at this locus suggests that it is intrinsically unstable.

The real function of Spade protein remains elusive. It appears to be conserved in sibling species of the *P. aurelia* complex (amino acid sequence identity with the *P. biaurelia* ortholog is 88%, and with that of *P. sexaurelia* ~79%). Judging by the presence of zinc-binding domain, the best conserved part of its sequence, and a coiled-coil domain detected in the encoded protein, it can be predicted to be a transcription factor [20]. However, coiled-coil domains are present in many proteins [21], including some involved in subcellular infrastructure maintenance [22]. One could speculate that the Spade protein may be a moonlighting protein, i.e., a protein which has a primary catalytic function but may also acquire a secondary non-enzymatic role, for example aldolase in *Plasmodium* [23] or transcription factors in mammals [24]. Although *Spade* expression increases during autogamy [9], when many genes become differentially expressed [25], it is evident that it plays an important role during vegetative growth. However, since *Spade* gene knockdown leads to quick changes in aberrant cell shape, and the recovery of a normal cell shape requires several divisions, it is conceivable that the protein is involved, at least indirectly, in submembrane cytoskeleton formation. Moreover, like all screwy mutants, Spinning Top is prone to form multinuclear monsters, i.e., cells which cannot accomplish cytokinesis, which also points to some acute problems with cytoskeleton or cortex. The phenomenon of cortical inheritance is well known in *Paramecium* [26,27]. It is thought to reflect the fact that aberrant association of monomers in multimeric proteins, leading to malformation of cytoskeletal filaments (or simply the absence of normal cytoskeletal structures), can be maintained during cell divisions despite continuous production of the proteins involved, much like prions. In the case of *Spade* knockdown and recovery, cells regain the normal shape only after several complete cell cycles. Determination of the intracellular localization of the *Spade* protein would help understand whether it may have a role in cortical or subcortical structure maintenance. As for the other screwy loci identified by previous genetic analyses (*sc66* and possibly *sc64*) [5], they might encode other proteins involved in cortical structures; if the *Spade* protein simply acts as a transcription factor, they may be genes that are transcribed in a *Spade*-dependent manner.

The pleiotropic effect of screwy mutations at the *sc1* locus can now be explained by the fact that these MIC deletions remove several genes. The *sc1-a* mutant has normal trichocysts, while the *sc1-c* mutants are characterized by an unusual, cigar-like shape of trichocysts [5,6] (Figure S4). This phenotype may thus be due to one or more of the 5 genes that are deleted in the *sc1-c* mutants, but retained in the *sc1-a* mutant. Consistently, the Spinning Top mutant, which also lacks these genes, has cigar-shape trichocysts.

It has long been known that alternative rearrangement patterns in the MAC, such as the deletion of a cellular gene in one cell line and its retention in another, are maternally transmitted across sexual generations in wild-type cells through both conjugation and autogamy. These maternal effects have led to the discovery of the scnRNA pathway, which regulates genome rearrangements during MAC development [2,3]. In this study we have shown that in crosses between screwy mutants and the wild-type cells, the absence of the *Spade* gene region in the MAC of the mutant parent does not result in the same deletion being reproduced on the wild-type allele that is introduced by conjugation in the mutant’s cytoplasmic lineage. This may seem surprising, but is in fact fully consistent with the current model for scnRNA action. Indeed, scnRNAs are initially produced from the entire MIC genome during meiosis in each conjugating partner, and are then thought to scan the parental MAC genome in each cell by pairing interactions with nascent transcripts; only those that cannot find a match, i.e., those produced from MIC-limited sequences, will then be licensed to target the elimination of homologous sequences in the developing zygotic MAC [28]. In the present case, the mutant parent cannot produce scnRNAs from the *sc1* locus during meiosis of the MICs, since these sequences are absent from its MIC genome. Thus, after introduction of the wild-type *sc1* allele from the wild-type partner, there are simply no homologous scnRNAs in the mutant cell to target deletions in the wild-type allele, which is therefore fully retained in the heterozygous F1 MAC. A very similar case has already been described, though this was long before the discovery of the scnRNA pathway. The d12 mutant carries a MIC deletion of the gene encoding surface antigen A, but when it is crossed to the wild type, the lack of that gene in the d12 parental MAC does not induce the developmental deletion of the wild-type allele in the MAC of its heterozygous cytoplasmic progeny [29,30]. This effect has so far remained mysterious, but can now be explained exactly in the same way.

**Figure 8:**
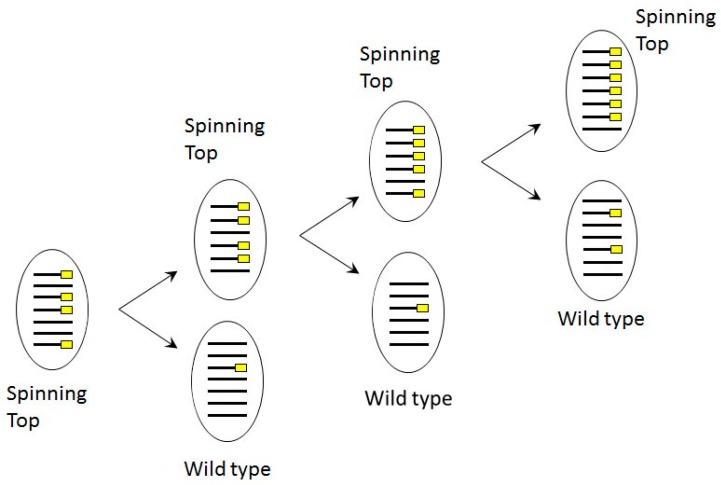
Scheme of copy number dependent phenotype segregation in phenotypically Spinning Top cell line, where MAC deletion was induced in *sc1* locus by dsRNA feeding in autogamy. Lines correspond to MAC chromosomes bearing the *Spade* gene, yellow rectangles indicate chromosome copies with deletions.

The induction of MAC deletions at the *sc1* locus by dsRNA feeding during autogamy always resulted in a broad spectrum of deletions of different sizes in each postautogamous clone, and most clones contained at least a fraction of wild-type MAC copies. The phenotypes of cells in each clone varied accordingly, from wild-type to pronounced screwy phenotype, and the ratios of phenotypes were probably revealing the ratios of wild-type and deletion-bearing MAC copies and their random fluctuation during amitotic divisions of the MAC (Figure 8). When the wild-type copy number increases over some threshold, the *Spade* gene dose becomes sufficient to produce enough protein for wild phenotype recovery. During vegetative growth, such clones always gave rise to phenotypically wild-type subclones, even when mutant phenotypes were selected for daily isolation.

Phenotypic reversion also occurred at a high frequency after autogamy of phenotypically mutant clones. Nevertheless, we were able to maintain them without selection for almost a year in stock tubes, and in all cases, we were still able to find both phenotypically mutant and wild-type cells in the cultures. Thus, induction of MAC deletions by dsRNA feeding during autogamy allowed us to obtain epimutants, as has been shown for other genes [19].

The appearance of screwy mutants has often been described in mutagenesis studies. Whittle and Chen-Shan [8] reported that cortical mutants had appeared under nitrosoguanidine treatment with a frequency of 1:1000, and two of eleven mutants they obtained were screwy mutants. Of all mutagenic factors which have led to screwy mutants in *P. tetraurelia* (see Table 1), only X rays can directly induce single and double strand breaks in DNA. Ultraviolet radiation induces formation of thymine dimers which later are repaired by nucleotide excision repair enzymes or by photoreactivation system; nitrosoguanidine is a well-known chemical mutagen which adds alkyl groups to guanine and thymine, which later can be repaired by the DNA mismatch repair system [31]. Furthermore, the Spinning Top mutant seems to have arisen spontaneously. Of course, we cannot exclude the possibility that this was somehow triggered by the heterozygous *mtGa-1* mutation in its parental clone [4], especially since similar phenotypes appeared on two other occasions during the genetic analyses of the *mtGa-1* mutant (S. Bhullar, unpublished data). Spinning Top deletion determines conspicuous phenotype, and it might be that other stealth deletions also occur under the influence of the *mtGa-1* mutation. However, whether the *mtGa-1* mutation may have a mutator effect remains unknown.

Such frequent occurrences of screwy-like mutations in different conditions, and the absence of the *Spade* gene in all available *sc1* mutants, suggest that the *sc1* locus is intrinsically unstable in the strain 51 MIC genome, and is a hotspot for some enigmatic chromosome breakage event. We have found at least 3 different deletion breakpoints among the mutant *sc1* alleles examined, but did not find any consensus or sequence motif which could be readily suspected to serve as a breakage site at these positions. The so-called common chromosome breakage sites in mammalian cells are often found in regions of the genome that are particularly sensitive to replication stress [32], increasing the probability of DNA damage in each cell cycle, and genome instability. Common chromosome breakage sites are known to be associated to loci containing many AT dinucleotides or minisatellite repeats which are also very AT-rich [33]. The *Paramecium* MIC genome is extremely AT-rich [34], as are the sequences around the mapped breakpoints. It may be also important that these sites are surrounded by IESs (Figure 4). The possible roles of IES density and AT richness in determining chromosome breakage hotspots in *Paramecium* remain to be elucidated; this would require identifying other examples of similar chromosome fragile sites.

## Figures and Tables

**Table 1:** *P. tetraurelia* mutants with screwy phenotype.

Mutant Strain Index */Strain of Origin	Original Mutant Name	Mutant Cell Line Index	Genotype	Obtained by	Origin of Mutation	Concomitant Phenotype Features	Independent Mutations in the Multiple-Marker Stocks
**Mutants studied in this work**							
scr1-1/51	sc	d4-136	*sc1-a*	D. Cronkite [5]	nitrosoguanidine	not documented	none
scr1-1ci big/51	sc	d4-75	*sc1-c^1^*	T. Sonneborn [5]	UV light	cigar-shaped functional trichocysts	big
1-3ci/d4-2	m1	-	*sc1-c^3^*	J. Beisson & M. Rossignol [7]	nitrosoguanidine	cigar-shaped functional trichocysts; slow growth	none
1-4ci/d4-2	m2	-	*sc1-c^4^*	J. Beisson & M. Rossignol [7]	nitrosoguanidine	cigar-shaped functional trichocysts; slow growth	ts401
1-8ci/d4-2	1-8ci	-	*sc1-c^8^*	M. Rossignol	UV light	cigar-shaped functional trichocysts; slow growth	none
Spinning Top/51	-	-	Spinning Top	S. Bhullar [4]	spontaneous mutation	not documented	none
**Other mutants documented but not available anymore**							
-/51	mut65	d4-85	*sc1-b*	C. Kung [8]	nitrosoguanidine	not documented	none
-/51	mut80		*sc1-d*	In genetic class [8]	nitrosoguanidine	not documented	none
-/51	1961-38-8	d4-137	*sc1-c^2^*	R. Kimball [5]	X rays	cigar-shaped functional trichocysts; cells large, spin less fast	none
-/51	1961-45-601	d4-137	*sc1-c^5^*	R. Kimball [5]	X rays	cigar-shaped functional trichocysts	none
-/51	-	d4-99	*sc1-c^6^*	D. Cronkite [5]	nitrosoguanidine	cigar-shaped functional trichocysts	none
-/51	-	d4-186	*sc1-c^7^*	S. Pollack [5]	X rays	cigar-shaped functional trichocysts	none
-/51	mut64	-	*sc64* **	A. Whittle [8]	nitrosoguanidine	normal trichocysts	none
-/51	mut66	-	*sc66* **	A. Whittle [8]	nitrosoguanidine	normal trichocysts; rotating clockwise when swimming	none

* as in [9]. ** Loci *sc1*, *sc64* and *sc66* are unlinked according to [5].

## Supplementary Materials

The following are available online at https://www.mdpi.com/2073-4425/10/7/513/s1, Figure S1: The origin of the Spinning Top mutant, Figure S2: Results of PCR demonstrating absence of the *Spade* gene in screwy mutants, Figure S3: Various deletions in the Spinning Top locus induced by dsRNA feeding, Figure S4: Shape of trichocysts in *P. tetraurelia* wild type and *sc1-c^3^* mutant cells. Table S1: Primers used for preparation of the feeding inserts, Table S2: Primers used for chromosome walking and for MIC-specific PCRs, Table S3: Genes from the MAC scaffold51_77 segment deleted in the Spinning Top mutant. Video S1: wild-type *P. tetraurelia* strain 51 phenotype, Video S2: Spinning Top mutant phenotype.
