# Supplementary material for: Loss of a Fragile Chromosome Region leads to the Screwy Phenotype in Paramecium tetraurelia
Source: Genes (Basel). 2019 Jul 6;10(7):513. doi: 10.3390/genes10070513 (PMC6679132; doi:10.3390/genes10070513)
Supplement: Supplementary file 1 [file genes-10-00513-s001.zip › Suppl figures and tables.pdf]

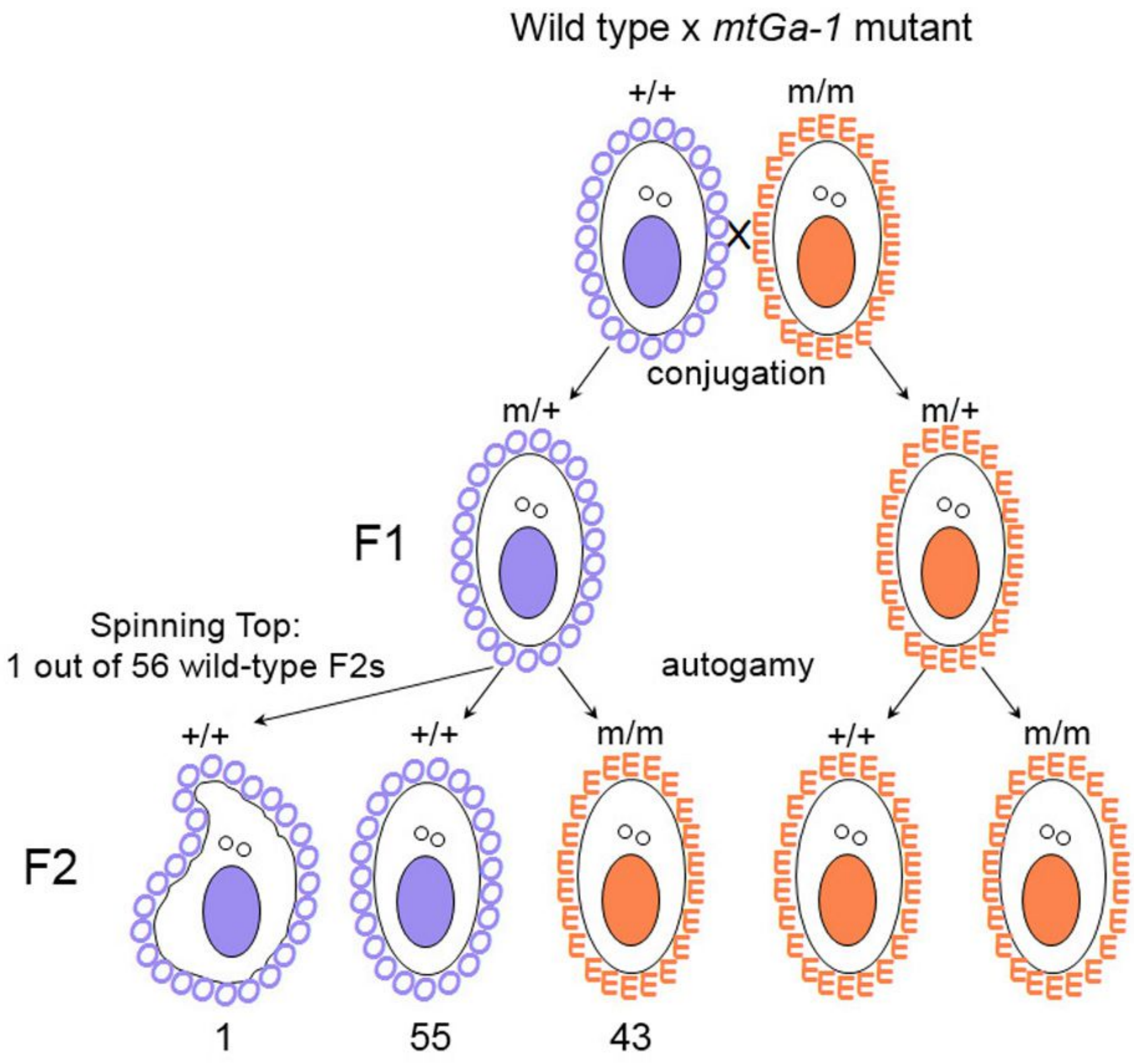

Supplementary Figure 1. The origin of the Spinning Top mutant (+ is wild type allele, **m** is a mutant allele of the *mtG* gene; see [4])**.**

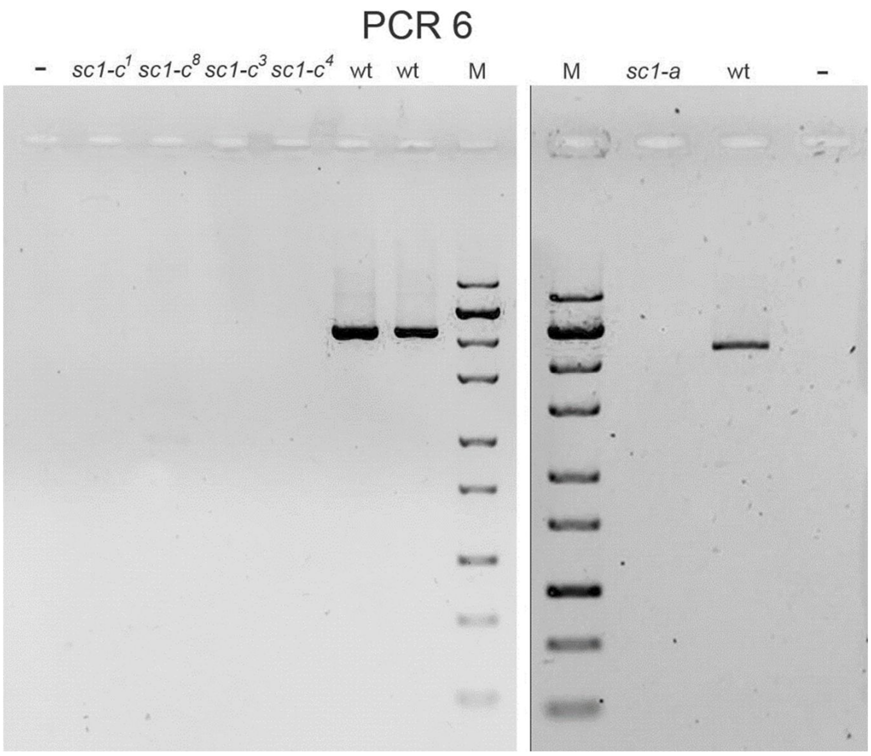

Supplementary Figure 2. PCR results demonstrating absence of the *Spade* gene in screwy mutants. The gene is present only in wild type cells (wt). "–" stands for negative control. For the primers used see Supplementary Table 1.

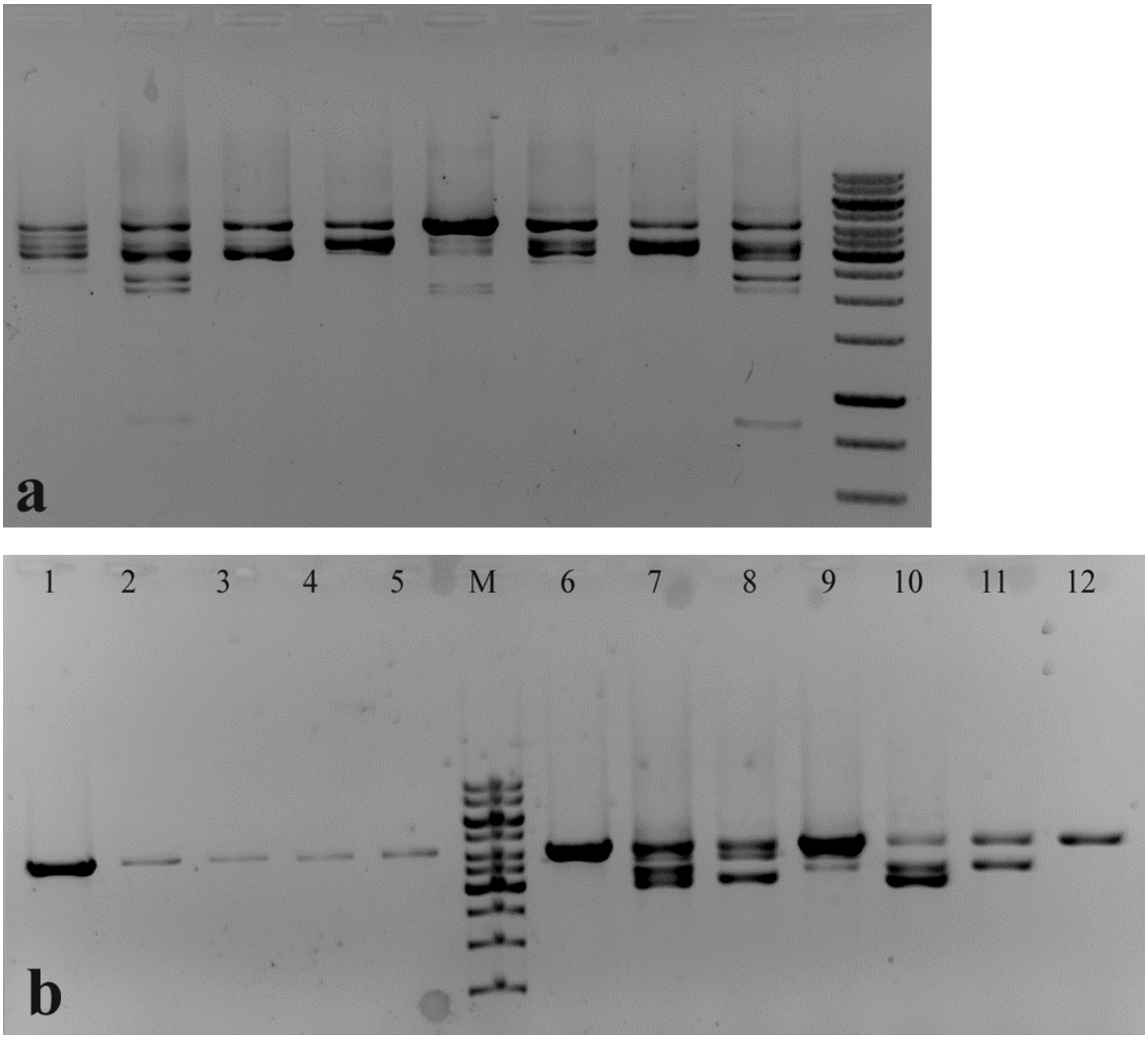

Supplementary Figure 3. (**a**) Results of PCR demonstrating various deletions in the *Spinning Top* locus induced by dsRNA feeding with the *Spade* gene fragment in autogamy. The fragment was the same as used for the *Spade* gene silencing. Deletions were detected by PCR using primers SPaD1 and SPaD2. (**b**) Results of PCR demonstrating that induced deletions can be inherited as epimutations through autogamy in lines of selected feeding-induced screwy cells (lanes 6-12, right to the marker, M). No deletions are present in lines obtained from phenotypically wild type cells from the same stocks (lanes 1-5). In some selected "screwy" lines deletions also disappear, and wild phenotype recovers (lanes 6, 12).

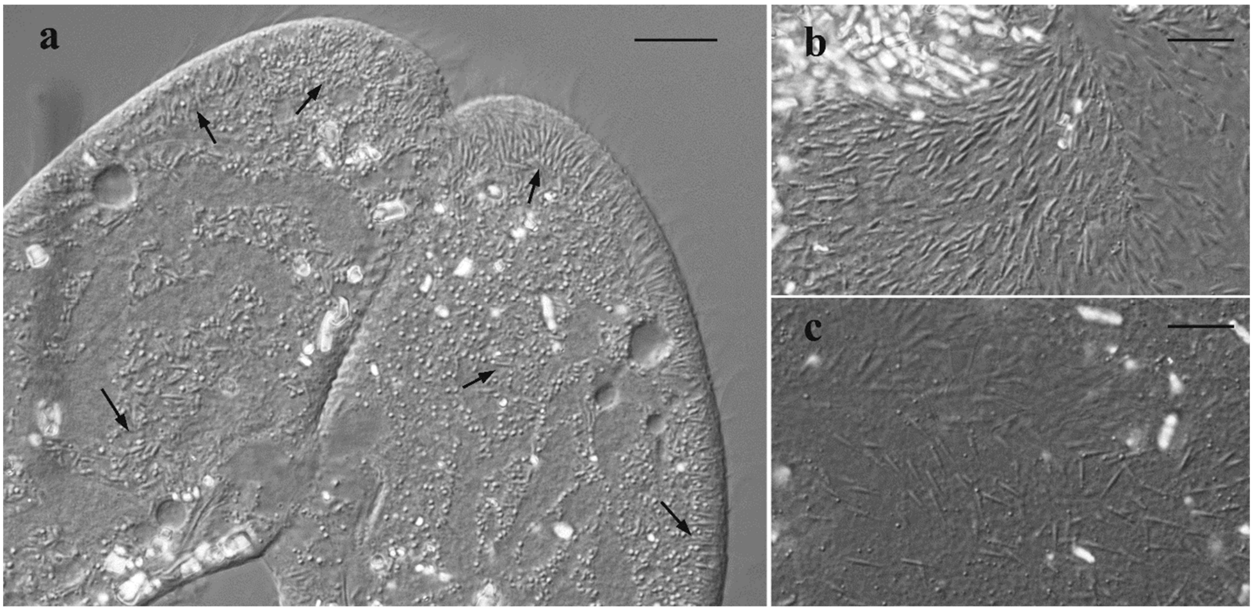

Supplementary Figure 4. Shape of trichocysts in *P. tetraurelia* wild type and *sc1-c*^{3} mutant cells. DIC microscopy. (**a**) Conjugating couple of wild type (left) and mutant (right) cells. Well-visible trichocysts are marked with the arrows. Scale bar is 5 µm. (**b**) Carrot-shaped (normal) trichocysts of the wild type cell. (**c**) Cigar-shaped (aberrant) trichocysts of the *sc1-c*^{3} mutant cell. Scale bar is 12 µm.

Supplementary Table 1. Primers used for preparation of the feeding inserts.

**Target gene**	**Gene index [9]**	**Primer index**	**Sequence**
«copine»	PTET.51.1.G0770200	Copine F	AGATGGATGGAATTATGCAAGCC
		Copine R	CTCCTTCCAAAGGAATCATGTAGAT
Spade	PTET.51.1.G0770206	fSpAD-F	TAATGGTACCTATTGAGCAGGAGGTTATTGT
		SpAD-R	GTACAACCCTGTCAAACCTTAAAC
"NimA"	PTET.51.1.G0770208	NIMA-F	CCACAGAGATATTAAGCCAGCC
		fNIMA-R	TTAAAGATCTGGCATTGGTCTTGCTTCAG
Spade WGD2 ohnolog scaffold 81	PTET.51.1.G0810029	SpAD81-F	TTTTGGTACCTATTAAACTGGAGCCTATTATTTAC
		SpAD81-R	ACCCAGATCTCCTATAAGATAGAC
Spade WGD2 ohnolog scaffold 94	PTET.51.1.G0940176	SpAD94-F	AACCGGTACCAAAAACCGCAACACAGTC
		SpAD94-R	GGGTAGATCTTCCACACCCTTCTGTA

Supplementary Table 2. Primers used for chromosome walking and for MIC-specific PCRs (see Fig. 2 for locations of the primers and for PCR design).

PCR	Forward primer		Reverse primer	
	Index	Sequence	Index	Sequence
PCR1	61F	GGAAGTGCCGAAACTACACTAGC	61R	GATTTCCAGAGCTTCTGTAATCCC
PCR2			59-r3	CATAATTCAATAGGATTTCTCCAACAGAC
PCR3	BP77F	ATACTGATTTTCGATTGTGATGTGACATTTG	77BPD1	GTACATAGGGAGGAGGTTACTTTGC
PCR4	SPaD1	TACATCGGACAATAGTGAGTCCTATAA	Sp-r1	CAACATATCCACCTGGACCACC
PCR5			IES 30-R	GAGTGATCCAACTATATCACCTTTCTTC
PCR6			SPaD-r	GTACAACCCTGTCAAACCTTAAAC
MIC-PCR			MICsp1-28R	TATATAGTTAAGCAACCCACAAAGAG
Induced deletions check (not shown in Fig. 2)			SPaD2	TTTCGAACTGTGGATCTTGATCATTC
PCR7	NIMA-1	TGCATTACCAAGCTCACTCGTC	NIMA-2	ACTTTCTAGCAAATAGGAACGGATCA
MIC-PCR positive control (not shown in Fig. 2)	77mic-f1	CCCCATCAAACCAGATAGTGCC	77mic-r1	GAATCGAGAACCGCACTCTACC

Supplementary Table 3. Genes from the MAC scaffold51_77 segment deleted in the Spinning Top mutant.

	**Gene index**	**Length**	**WGD1 ohnolog**	**Putative description and molecular function** (according to [9])	**Possible function** (according to [9])
1	PTET.51.1.G0770199	863 bp	Yes	Cytochrome b5-like Heme/Steroid binding domain	heme binding
2	PTET.51.1.G0770200	2402 bp	Yes	C2 domain; Copine	Copine
3	PTET.51.1.G0770201	1642 bp	Yes	Coiled coil domain	putative transmembrane protein
4	PTET.51.1.G0770202	1237 bp	No	Coiled coil domain	putative metallo-beta-lactamase protein
5	PTET.51.1.G0770203	982 bp	Yes	Aquaporin-like, membrane transporter	Major intrinsic protein (MIP) superfamily, aquaporin
6	PTET.51.1.G0770204	2553 bp	Yes	Cyclic nucleotide-binding-like, zinc ion binding, nucleic acid binding	Cyclic nucleotide-gated cation channel
7	PTET.51.1.G0770205	1583 bp	Yes	Pyridine nucleotide disulphide reductase class-I signature, oxidoreductase activity	Oxidation-reduction process, cell redox homeostasis
8	PTET.51.1.G0770206	1818 bp	No	Coiled coil domain, zinc-finger of a C2HC-type	Transcription factor
9	PTET.51.1.G0770207	1758 bp	No	Protein kinase-like domain, ATP binding, calcium ion binding	calcium-dependent protein kinase
10	PTET.51.1.G0770208	1729 bp	No	Protein kinase-like domain, ATP binding	Protein kinase, Nek family
11	PTET.51.1.G0770209	891 bp	No	Nucleotidyltransferase family	protein phosphatase
12	PTET.51.1.G0770210	1038 bp	No	Mitochondrial substrate/solute carrier	Mitochondrial substrate carrier protein, inner membrane transporter
13	PTET.51.1.G0770211	616 bp	Yes	Small GTPase Rab1 family profile	small GTPase, Rab type
14	PTET.51.1.G0770212	1064 bp	Yes	Translation initiation factor 2, alpha subunit, RNA binding	Eukaryotic translation initiation factor 2 subunit 1
15	PTET.51.1.G0770213	1281 bp	No	Heat shock transcription factor family, sequence-specific DNA binding	DNA-templated transcription regulator
16	PTET.51.1.G0770214	968 bp	No	Not annotated	Not annotated
17	PTET.51.1.G0770215	335 bp	No	Hypothetical protein	Not annotated
18	PTET.51.1.G0770216	705 bp	No	Transmembrane domains	Not annotated
